# Operative Dentistry, or Filling Teeth

**Published:** 1871-10

**Authors:** C. M. Wright


### OPERATIVE DENTISTRY, OR FILLING TEETH.

BY PROF. C. M. WRIGHT.

Read before the Mad River Dental Society, September 12, 1871.

Perhaps friends may think that the writer of this paper should let his pen follow other paths. He has never distinguished himself as an operative dentist—has never described his method—has never elaborated a filling with a fluent tongue before this or any other society—has never had figures of *his* pattern of pluggers published in the advertising columns of a dental periodical—has never been called upon by a *god* in the profession, at a national association, to "tell them how you do it Willie, or Charlie, or Sammie"—using the christian name as a mark of special recommendation, approbation and admiring familiarity.

These things give a man the right to speak with authority on the subject. These give what we prize so high—fame. In fact, this is fame. The demi-god A. says, "that man can fill teeth better than any other dentist in the country," and all the balance of the alphabet go on saying "that man can fill teeth better than any other dentist in the country," without ever having seen a single operation of the favored one, or knowing for themselves anything about the matter. And so it goes in the medical profession, and so it goes in the dental profession. What is operative dentistry? It is the surgical (that word is more genteel than mechanical) operation of cutting away decomposed and decomposing dentine and enamel of teeth—cutting the cavities into good shape for retaining foreign substances, and then stuffing, stopping, plugging or filling the cavity, as well as possible with gold, tin, amalgam, or oxychloride of zinc. This operation, when performed neatly, rapidly and skillfully, without causing extra pain, is as fine a surgical and mechanical operation as we know of. It requires but a very short time for a man of ordinary ability, to learn to fill a cavity in a tooth, fastened in a vise, but it takes labor and practice to learn to perform this operation, when the cavity and tooth are found in an unfavorable location in a nervous patient's mouth. The difficulty of the operation makes it a more uncertain mechanical success. At different periods, and in different localities, even in our own country, entirely different modes of performing this stopping, stuffing or plugging operation, have been and are the fashion. For instance, the writers of twenty or thirty years ago, describe a method of stuffing gold into cavities entirely different from the methods we to-day consider good. Throughout the south, we find elegant operators who use soft or non-adhesive gold entirely, introducing it in the form of pellets, cylinders, balls, strips and ropes, who do not understand or believe in the northern and more fashionable, (because more modern) mode of using adhesive gold, and depending upon the adhesion or cohesion of little pellets or mats of gold, to build their cavities full of the precious metal.

In Ohio, nearly every dentist fills after the latest fashion. Day before yesterday the only way to fill teeth, was to roll Nos. 4 or 6 adhesive gold in a napkin, cut the strips so rolled into pellets, start from a retaining pit and tack on pellet by pellet, the gold. Yesterday heavy foils, heavy mallets, and rubber dams were the fashion. To-day, smooth pointed pluggers rage—he who does not adopt the style in vogue, is gently dropped into the oblivious past, and can not sit down at the round-table with choice spirits who are in the "advance"

Many a poor, weak-minded brother, does not possess the moral courage to stay in the rear. It tickles him to have an *advance* man smile on him. It fills his mind with a black gloom to think that the advance may turn coldly from him—not recognize him. This is not simply rhetoric or talk. I am not writing this because I want to write—I am stating a terrible fact—and I received it from the "fount of inspiration," as truly as you or anybody else ever received instruction from that source. But what are we striving after in our advance movements? Is the advance guard moving in the right direction? Are the leaders of the advance infallible? Ask them if they are saving more teeth by their present modes—if they are gaining time in their operations—if they are simplifying the operation of filling teeth—if they are lessening the pain and discomfort of the operation for their patients? The rear guard cry No! Ohio dentists are spending their lives (and their lives are not the lives of mere mechanics—Oh no!)—are spending their lives I say, in the noble occupation of trying to perform difficult mechanical operations on the teeth. Given a plain cavity in an inferior molar, easy of access, healthy patient, everything favorable—the cavity moderately large, requiring from twelve to fifteen or eighteen grains of gold to stop it. What do we do? 1st. Spend from ten minutes to one hour in arranging, with considerable pain, much mouth stretching, and some gagging to the patient, a rubber coffer dam. 2nd. Spend from one to two or three hours in tacking on small pellets of gold, layer by layer from a retaining pit, with a muscular boy and a steel or lead mallet for our power. 3d. We trim, and burr, and file and polish—what have we accomplished? A beautiful operation—solid gold in a hole in a tooth. We have also tired ourself, tired our boy with his sledge-hammer, and if the patient is not a cast iron man, have almost killed him. Every body feels strained. Every body needs rest. Every body feels that two or three efforts like this would be enough for one day. Three holes in the teeth stopped—enough for one day's work! The old fashioned operator dried the cavities placed a little napkin about the tooth, and filled the cavity with cylinders or plugs of soft gold in from five to twenty minutes, with much less fatigue, inconvenience and pain to the patient, and a shorter muscular exercise for himself, and the plug saved the tooth twenty or thirty years. Let us look at the thing mechanically. You have a hole to stop in a plank. Will you take saw dust and little chips and lay them in and fasten each fragment there one by one, when the water is rising rapidly and may flood your work? Or will you select a plug and drive it in, stopping the hole at once?

But teeth are not planks. No, but the principles of common sense and mechanics are good, and can be applied in both cases. "Oh!" I hear you say as you said about opinions on another subject of the same writer. "He has low ideas about the duties of his profession." "He hasn't progressive notions." "He is behind the times—the older dentists among us used to fill teeth years ago in some such way and now when we look back we think that then we didn't know how to fill teeth at all." Well, you have a right to opinions, if they are carefully formed, and yet every old dentist among you has confessed, when chance has thrown in his way one of his early operations, "By George, those fillings have kept well, and kept the teeth well too."

I claim that it is unprofessional to spend half a day in performing an operation that can be done as well, with more certainty of success in stopping a cavity and arresting decay, in twenty minutes. I say to the dental profession, you are spending too much time, too much mechanical skill, too much labor, too much patience for the amount of real work you do—for the number of teeth you try to save. You are degenerating from a high and noble professional position, to that of simply *skilled mouth jewelers.* All we want to accomplish as dental surgeons, is the arrest of the disease, caries, and the restoration of an organ to usefulness and health. Hard, beautifully compact and highly polished lumps of gold set in a tooth do not always do this. It requires great patience and time and skill to adapt this hard gold perfectly to the unequal walls of a cavity—greater skill, more patience, longer time to each operation than the majority of us can at all times command.

With soft gold, and the old way of filling, nearly all of us can stop a cavity. I think a much larger per cent. of teeth have been, are and can be saved for usefulness and time, by the shorter and more philosophical way, aside from the advantages of time gained, the greater number of teeth that can be cared for with the same expenditure of skill and patience, strength and comfort. Many of the finest operations of our best operators fail, while the gold itself is capable of lamination. This is unfortunately a very serious *fact.*

Perhaps I am wrong in my opinions. Any of us may be mistaken in our favorite opinions, but I do not wish to be apologized for by my friends. The opinions expressed here are based on considerable thought, a good deal of careful observation and some experimenting, all bearing on this subject of filling teeth. They are also opposed to early instructions received. But if I stood alone on the question, I should have to brave your condemnation, and express my belief that I think we need reform in this department of dentistry. We need a little retracing of steps. We need go backwards a short distance. I am not alone in these views, however, but have the countenance of all but the advance guard and those who are led by them. Because adhesive gold is good and valuable in certain cases, is no reason why we should make a large proportion of our operations *capital*, when they would be *miner* if we employed soft foils.

At the meeting of the American Dental Association, at Nashville, Dr. Forbes excused himself for not being able to make himself heard, on the ground of having lost his teeth by "first-class dentistry." Thousands of mouths are in like condition. At the same meeting a paper was read by Dr. Knapp, of New Orleans, on "The Saving of Time in Dental Operations." It is a valuable paper, and well worth the attention of our slow-coach Ohio brethren. Another point might be urged on this topic, though it will not be expected that the subject shall be fully elaborated in a paper of this kind. It is the question of fees to the dentist and expense to the patient. The man who takes three hours to fill a tooth, should receive, by a law in political economy, as much for that one filling, as he should get for three, who fills three teeth in three hours. The slow method, therefore, tends to increase the price of an operation, the price of a tooth. Is there any advantage, or any object to be gained in raising the charges for saving a tooth threefold? Not if we are philanthropists as well as physicians and surgeons of the mouth. Unless our philanthropy embraces only the dentist·

The greatest good to the greatest number is a good rule in operative dentistry. A great many men who have come into the profession within ten years, in our state, have never seen a soft foil filling, and do not know the advantages. To them I would say, try it in your laboratory, try it in simple crown cavities, learn the principle and test it for yourselves. Prepare your cavity without retaining pits—make cylinders, block or pellets a little longer than the cavity is deep. Set them in the cavity and apply lateral pressure against the walls of your cavity; wedge them in with a key block or cylinder—consolidate the surface with the burnisher, and finish. Or stuff your cavity full of gold, solidly against the walls in any way most convenient to yourself and patient, and the way you can do it quickest and best. Do this well. You can conserve teeth, avoid unnecessary pain, and fill twenty teeth per day (if the cavities are in favorable position) when you become expert. Reserve your adhesive foil for special cases. Think over this brethren, and give the writer credit for candor, at the risk of his reputation in the State of Ohio, as an operative dentist.

